# Volleyball practice increases bone mass in prepubescent boys during growth: A 1-yr longitudinal study

**DOI:** 10.1371/journal.pone.0266257

**Published:** 2022-04-07

**Authors:** Anis Zribi, Hamada Chaari, Liwa Masmoudi, Wajdi Dardouri, Mohamed Ali Khanfir, Elyes Bouajina, Monia Zaouali, Mohamed Zouch

**Affiliations:** 1 Research Laboratory of Exercise Physiology and Pathophysiology: From Integral to Molecular "Biology, Medicine and Health" (LR19ES09), Faculty of Medicine of Sousse, University of Sousse, Sousse, Tunisia; 2 Research Laboratory: Education, Motricity, Sport and Health, EM2S, LR19JS01, High Institute of Sport and Physical Education of Sfax, University of Sfax, Sfax, Tunisia; 3 Department of Sport Sciences and Physical activity, College of Education, University of H’ail, Hail, Saudi Arabia; 4 Department of Rheumatology, FarhatHached Hospital, Sousse, Tunisia; eCampus University, ITALY

## Abstract

The aim of this longitudinal study was to examine the effects of 1-yr of volleyball practice on the bone mass development in the growing skeleton among prepubescent children. Twenty volleyball players and nine teen matched control boys (Tanner stage 1, at the start of the study) were followed over a 1-yr period. Bone mineral density (BMD, g/cm^2^), bone mineral content (BMC, g) were measured by dual-energy X-ray absorptiometry on the whole body, lumbar spine (L2–L4), legs, arms, femoral necks, hips and radii. At follow-up, in comparison with controls, volleyball players gained more BMD in whole body (4.5% *vs* 1.7%; p = 0.014), both nondominant and dominant arms (5.8% *vs* 1.1% p = 0.005, and 6% *vs* 2.1%; p = 0.003, respectively), both nondmoninat and dominant legs (9% *vs* 4.8%; p = 0.005 and 10.7% vs 6% p = 0.0025; respectively), dominant ultradistal radius (10.4% v*s* 0.9%; p = 0.005), dominant third distal radius (9.6% vs 3.71%; p = 0.023), dominant whole radius (7.4% *vs* 3.1%; p = 0.017), lumbar spine L2-L4 (9.9% *vs* 2.8%; p = 0.004), femoral neck (4.7% *vs* 1.6%; p = 0.034), trochanter (6% *vs* 1.5%; p<0.001) and total hip (6.1% *vs* 2.6%; p = 0.006). Volleyball players gained more BMC in both nondominant and dominant arms (25.1% *vs* 13.4%; p = 0.003, and 26.1% *vs* 15.6%; p<0.001 respectively), both nondominant and dominant legs (20.2% *vs* 14.5%; p = 0.004 and 23% *vs* 16%; p = 0.004, respectively), dominant ultradistal radius (22.4% *vs* 8.7%; p = 0.002), dominant third distal radius (20.9% v*s* 5.9%; p = 0.001), dominant whole radius (20% *vs* 13%), nondominant third distal radius (14.5% *vs* 5.9%; p = 0.001), nondominant whole radius (21.1% *vs* 12%; p = 0.002), lumbar spine L2-L4 (21.1% *vs* 13.7%; p = 0.007), femoral neck (25.9% vs 8.7%; p = 0.007), trochanter (23.5% *vs* 17.1%; p = 0.006), and total hip (16.3% *vs* 11.3%; p = 0.009) than controls. A close correlation was observed between the increment (Δ) of whole body lean mass and increased (Δ) BMD and BMC in whole body (r = 0.43, p<0.01, r = 0.73, p<0.001; respectively), lumbar spine (r = 0.54, r = 0.61, p<0.001; respectively), trochanter (r = 0.46, p<0.01, r = 0.35, p<0.05; respectively), and total hip (r = 0.53, p<0.01, r = 0.6, p<0.0001; respectively). In summary, 1-yr of volleyball practice has an osteogenic effect on bone mass in loaded sites in prepubescent boys.

## Introduction

Low bone mineral density (BMD) is the major determinant for osteopenia and osteoporosis, which increase the chances of fragility fracture and bone injury, particularly in the radius, hip and spine [1]. However, it is becoming increasingly apparent that the antecedents for osteoporosis begin in childhood [2]. Establishing an optimum level of bone mineral in childhood and adolescence is essential to offset the inevitable loss of bone in later life [3].

Previous research has shown positive effects of physical activity on bone mineral accrual, especially when practiced before [4, 5] or during puberty [6, 7]. Physical exercise (particularly weight-bearing activities) is a particularly relevant factor for maximizing bone mass, and widely recommended as one of the key preventive strategies to reduce the risk of osteoporosis, falls and fractures [8].

The variable mechanical stress induced through physical activity is the stimulus for the related increase in bone formation [9]. The skeletal response to exercise is therefore, dependent on the nature of the loading forces associated with the activity [10]. Indeed, during growth, athletes, who participate in weight-bearing activities, have higher bone mineral density (BMD) than sedentary controls [11]. Zouch et al, found among early pubertal soccer players an increase in bone mineral content (BMC) in whole body and weight-bearing bones (lumbar spine, total hip, and supporting leg) and non-weight-bearing bones (dominant arm and non-dominant arm) compared to controls [12].

Furthermore, high-impact sports are associated with greater osteogenic benefits than low-impact sports [10]. However, the intensity of physical exercise can also influence bone gain. Prepubescent children engaged in high intensity weight-bearing activities such as soccer and basketball have higher bone mass when compared with children involved in low-intensity of these weight-bearing activities [13, 14].

Volleyball is a weight-bearing intermittent team sport characterized by quick and short displacements and vertical jumps, in either defensive and offensive actions [15]. It involves a number of different actions such as running accelerations and decelerations, rapid directional changes and repetitive jumping. Upper extremities are also sollicited in serving, passing, spiking and blocking the ball. All those actions are known to generate high strains stimulus at the upper and lower limbs by the reaction forces produced by the jumps, that amount to three to six times the body weight [16].

It has been reported that prepubescent volleyball players displayed greater BMC in lumbar spine, total hip and radius, and promoted more additional gains in the forearm and legs bone area than controls [17]. Similarly, female adolescent volleyball players (16.20±0.77 years) displayed higher values of BMC and BMD in the whole body, trunk, and lower limbs in comparison with swimmers [18]. Alfredson et al. reported that, young female volleyball players aged (20.9 +/- 3.7 years) and trained for about 8 hours/week, had ahigher BMD of the total body (6.1%), lumbar spine (13.2%), femoral neck (15.8%), Ward’s triangle (17.9%), trochanter (18.8%), nondominant femur (8.2%), and humerus (dominant 9.5%, nondominant 10.0%) compared with controls [19]. Therefore, volleyball may be considered as an osteogenic sport for prepubescent [17], adolescent [18, 20, 21], and adults [19, 22, 23]. In our knowledge, the effects of practicing volleyball in growing boys has not been studied. Therefore, the aim of this longitudinal study was to examine the effects of 1-yr of volleyball practice on the bone mass development in the growing skeleton among children. We hypothesized that prepubescent children practicing volleyball for 1-yr would have greater enhancement in bone mass in loaded bones than control subjects.

## Materials and methods

### Population

Thirty nine voluntary healthy boys, aged 10–13 yr (Tanner stage 1) and recuired from a selection of regional volleyball team of the Tunisian Sahel, were included in this longitidunal study which begins at the end of a sport period (June) and ends at the next sport period (12 months later). They were divided into 2 groups depending on their physical pattern: the volleyball group (VB) was composed of 20 voleyball players, belonging to the same selection of regional team, and who practiced volleyball for at least 18 months in addition to 2 weekly physical education sessions at school (of 50 min each). Volleyball players completed 4–6 hours of training plus one competition game a week. The other, 19 were assigned to the control group (C) who only had physical education at school and did not participate in any kind of sport during the previous 6 yr.

In general, volleyball training sessions lasted for 1h and 30 min, including about 15–20 min of warm-up, low-intensity games and stretching exercises, 10–25 min of technical volleyball exercises characterised by explosive actions such as lateral movements, passing actions, blocking, jumping, setting, serving, and spiking, 20–30 min of mini-volleyball match practice, and 10 min of active recovery.

Boys who have chronic diseases that might affect bone metabolism were automatically excluded from the study. This study was approved by the Independent Ethics Committee of Farhat Hached Tunisian Hospital and had been led according to the World Health Organisation’s recommendation elaborated at Helsinki [24]. All children’s parents were asked to read and sign an informed consent document before participation.

#### Anthropometric measurements

Height was measured to the nearest 0.001m using a wall mounted stadiometer (model S-220; Seca, Hanover, MD), and weight was assessed to the nearest 0.1 kg using a Seca electronic weighing scale (model 770; Seca). Body mass index (BMI, in kg/m^2^) was calculated as follows: BMI = Weight/Height^2^.

#### Calcium intake

The amount of calcium consumed per day (mg/day) was measuredby using the Bilnut SCDA Nutrisoft (Cerelles, France) program, a method of recording food for 3 consecutive days.

#### Bone measurement

BMD (g/cm^2^) and BMC (g) for the whole body; lumbar spine (L_2_-L_4_); femoral neck of the dominant leg; dominant and nondominantradius; lean mass (kg); and fat mass (kg) were measured by dual-photon absorptiometry X-rays by DXA (Lunar Prodigy, model DXAP 2004, Madison, WI, USA, software version 3.6).

#### Parameters of physical activity

*Aerobic maximal power (VO*_*2*_*max)*. The maximum oxygen uptake (VO_2_max) was estimated by the hrough the 20-m shuttle run test of Leger et al. [25].

*Basal physical activity level (PAL)*. Basal physical activity level was calculated by using the Bratteby’s questionnaire which estimates the level of daily physicalactivities during a typicalday, without volleyball training. The subjects were asked about their various activities during 24 h. The day quantified was divided into 96 periods of 15 min. Each activity was categorized into nine levels according to their average energycosts (physical activity ratio (PAR)). The PAR values were ranged from 0.95 for sleep or rest in bed to 15 for manual work or maximal sport activity. The activity records were calculated by summing up the number of 15-min periods of each categorical activity levels. These results were then multiplied by the PAR value of each category to predict total energy expended (TEE).

Physical activitylevel (PAL) was then calculated using the formula PAL = TEE (in megajoule/day) / BMR (in megajoule/day) where BMR is basal metabolism rate and was predicted from age and body weight using the prediction formula, BMR = 0.0746×kg BW+2.754 MJ/ day [26].

*Peak power of lower limbs*. The peak power of lower limbs was evaluated by squat jump (SJ), counter movement jump (CMJ) (using Sargent test) and horizontal jump (HJ) (using a dual Dkm) [27].

*Pubertal status*. Tanner pubertal status was determined by serum rates of follicle-stimulating hormone (FSH), luteinizing hormone (LH), and testosterone [28] and confirmed by a clinical method of recognized validity and reability of Tanner [29]. We measured selected children twice for this analysis who were Tanner stage 1 at baseline and Tanner stage 2 at follow-up. Tanner stage1is noted as prepubescent, with no signs of secondary sexual characteristics. Tanner stage 2 is noted as early-puberty. The early puberty group was considered as the pubescent group [29].

### Statistical analyses

Statistical analyses were performed using STATISTICA Software (version 12, 2004, France). The data were expressed as mean ± standard deviation (SD). The variation between baseline anf follow-up (Δ) was calculated as follows: Δ = Follow up–Baseline.

The percentage of variation between baseline and follow-up is calculated by the following formula: Δ% = (Δ / Baseline) X 100.

After normality verification with the Shapiro-Wilk’s w test, parametric tests were performed.

The analyses of covariance entering height, weight and whole body lean mass as covariates were performed to evaluate differences in BMD and BMC between the two groups.

Mixed two-factor variance analyses (ANOVA) were used to verify the effect of independent variables (Groups and Training) on each of the study-dependent variables. These ANOVAs took the following form: 2 Groups (Controls vs.VB players) × 2 Training (Baseline vs. Follow-up). When (ANOVA) shows significant interaction, a Bonferroni post hoc test was applied to compare the averages two to two.

Effect sizes were calculated as partial eta-squared (η_p_^2^) to estimate the meaningfulness of significant findings.

Additionally, bivariate correlation analysis was applied to identify the relationship between the increment (Δ) of whole body lean mass, and the increment (Δ) of bone mass variables.

Statistical analyses were performed using Statistica software (Statsoft, version 12, 2014, France). Statistical significance was set at *p* ˂ .05.

## Results

### Anthropometric parameters, calcium intake, physical fitness and pubertal status

The subject’s age, anthropometric parameters, calcium intake, physical fitness and pubertal status at baseline and follow-up are summarized in Table 1.

**Table 1:** Anthropometric parameters, calcium intake, physical fitness, and pubertal status at baseline and follow-up (Mean± SD).

Parameters		Means±SD		Group × Training interaction		
		Baseline	Follow-up	F_(3,108)_	P-value	η_p_^2^
Age (year)	Controls	11±1	12±1#	0.765	0.387	0.020
	VB players	11±1	12±1#			
Height (m)	Controls	1.45±0.05	1.51±0.06#	2.836	0.101	0.071
	VB players	1.52±0.06*	1.59±0.07*#			
Weight (kg)	Controls	34.63±4.4	39.47±5.77#	0.293	0.592	0.008
	VB players	41.15±7.05*	45.6±7.64*#			
BMI (kg/m2)	Controls	16.5±1.63	17.34±1.73#	4.132	0.05	0.100
	VB players	17.7±1.97	17.9±1.74#			
Whole body FM (g)	Controls	4842±2175	5788±3166#	1.245	0.272	0.033
	VB players	5823±4356*	6332±3702*#			
Whole body LM (g)	Controls	27014±3385	30201±3873#	6.274	0.017	0.145
	VB players	31415±4032*	36091±5651*#			
Calcium intake (mg/day)	Controls	714.2±41.4	786±61#	1.044	0.314	0.027
	VB players	705.6±36.8	800.1±76.3#			
PAL (a. u)	Controls	36.1±1.8	37.9±1.9#	0.005	0.943	0.000
	VB players	37.1±2.6	38.9±2.2#			
VO2max (ml/kg/min)	Controls	47.2±3.6	47.6±2.6	10.341	0.003	0.218
	VB players	50.9±3.1*	53.5±2.7*#			
SJ (cm)	Controls	20.2±2.8	21.1±3#	115.867	<0.001	0.758
	VB players	26.7±3.4*	31.9±3.3*#			
CMJ (cm)	Controls	22.4±2.7	23.7±2.7#	82.465	<0.001	0.690
	VB players	30.3±4.1*	35.8±3.7*#			
HJ (cm)	Controls	155.2±3.2	162.9±4.4#	65.800	<0.001	0.640
	VB players	166.7±7.3*	192.8±8.4*#			
FSH (mUI/mL)	Controls	3.28±1.2	4.67±1.25#	1.268	0.267	0.033
	VB players	3.78±1.31	4.87±1.22#			
LH (mUI/mL)	Controls	2.08±1.08	3.42±1.38#	3.423	0.072	0.085
	VB players	2.09±0.93	3.83±1.4#			
Testosterone (mUI/mL)	Controls	0.27±0.16	2.46±1.67#	0.022	0.883	0.001
	VB players	0.28±0.17	2.54±1.17#			

**BMI**, body mass index; **CMJ**, Countermovement jump; **FSH**, follicle-stimulating hormone; **HJ**, Horizontal jump; **LH**, luteinizing hormone; **PAL**, physical activity level; **SD**, standard deviation;**SJ**, Squat jump; **VB players**, volleyball players; **VO2max**, maximum oxygenuptake (mL/kg/min);

* Significantly different from Controls at p<0.05;

^**#**^, Significantly different from Baseline at p<0.05.

No difference in age, BMI, calcium intake, physical activity level, FSH, LH and testosterone rates was observed at baseline and follow-up between the 2 groups. However, volleyball players were taller, heavier, and had higher lean and fat mass in whole body than the controls at baseline and follow-up. Furthermore, volleyball players showed better performances in all physical fitness tests at the 2 sessions of measurments (Table 1).

### Bonemeasurement

#### Baseline measurements

At baseline, after adjustement for heigh, weigh and whole body lean mass, scans did not reveal any significant difference in BMD between groups in all measured sites (Table 2). Similarly, no difference in BMC between the 2 groups in most sites except in whole body, dominant arm, both dominant and nondominant leg, and dominant whole radius, which was higher in volleyball players than controls. (p<0.05) (Table 3).

**Table 2:** Mean ± SD BMD (g/cm2) values for controls and volleyball players at baseline and follow-up.

Parameters		Means±SD		Group × Training interaction		
		Baseline	Follow-up	F_(3,108)_	P-value	η_p_^2^
Whole body	Controls	0.940±0.040	0.960±0.040	6.635	0.014	0.152
	VB players	0.940±0.060	0.980±0.060#			
Nondominant arm	Controls	0.690±0.050	0.690±0.030	4.099	0.050	0.100
	VB players	0.670±0.060	0.710±0.050#			
Dominant arm	Controls	0.690±0.030	0.700±0.030	9.999	0.003	0.213
	VB players	0.700±0.050	0.740±0.050*#			
Nondominant leg	Controls	0.960±0.060	1.010±0.050#	8.711	0.005	0.191
	VB players	1.000±0.090	1.080±0.090*#			
Dominant leg	Controls	0.960±0.060	1.020±0.060#	5.462	0.025	0.129
	VB players	1.000±0.100	1.100±0.100*#			
Dominant ultradistal radius	Controls	0.276±0.025	0.278±0.034	8.876	0.005	0.193
	VB players	0.275±0.040	0.302±0.040#			
Dominant third distal radius	Controls	0.486±0.042	0.503±0.044	5.588	0.023	0.131
	VB players	0.479±0.058	0.522±0.056#			
Dominant whole radius	Controls	0.381±0.028	0.392±0.034	6.247	0.017	0.144
	VB players	0.383±0.036	0.410±0.037#			
Nondominantultradistal radius	Controls	0.274±0.033	0.288±0.033	0.627	0.434	0.017
	VB players	0.262±0.044	0.283±0.041#			
Nondominant third distal radius	Controls	0.485±0.047	0.505±0.052	3.912	0.055	0.096
	VB players	0.463±0.065	0.507±0.063#			
Nondominant whole radius	Controls	0.378±0.033	0.395±0.032	0.508	0.481	0.014
	VB players	0.368±0.043	0.391±0.051#			
Lumbar spine L2-L4	Controls	0.750±0.060	0.770±0.060	9.550	0.004	0.205
	VB players	0.780±0.110	0.860±0.110*#			
Femoral neck	Controls	0.900±0.090	0.910±0.090	4.843	0.034	0.116
	VB players	0.930±0.110	0.980±0.110#			
Trochanter	Controls	0.740±0.070	0.750±0.080	17.788	<0.001	0.325
	VB players	0.740±0.100	0.790±0.110#			
Total hip	Controls	0.910±0.090	0.930±0.080#	8.498	0.006	0.187
	VB players	0.900±0.110	0.950±0.110#			

**VB players**, volleyball players;

* Significantly different from Controls at p<0.05;

^#^ Significantly different from Baseline at p<0.05

**Table 3:** Mean ± SD BMC (g) values for controls and volleyball players at baseline and follow-up.

Parameters		Means±SD		Group × Training interaction		
		Baseline	Follow-up	F_(3,108)_	P-value	η_p_^2^
Whole body	Controls	1358±142	1513±172	3.145	0.082	0.058
	VB players	1539±214*	1797±269#			
Nondominant arm	Controls	68.40±15.30	76.70±15.80#	9.799	0.003	0.209
	VB players	77.80±17.60	96.30±20.90*#			
Dominant arm	Controls	65.10±11	74.90±11.80#	18.969	<0.001	0.339
	VB players	79.80±16.80*	100.30±21.40*#			
Nondominant leg	Controls	252.80±38	289.20±41.90#	12.982	<0.001	0.260
	VB players	307.40±53.30*	368.70±65.90*#			
Dominant leg	Controls	253.10±39.90	293.60±47.50#	9.330	0.004	0.201
	VB players	306.70±56.80*	374.40±64.80*#			
Dominant ultradistal radius	Controls	0.78±0.08	0.84±0.10	9.636	0.004	0.207
	VB players	0.86±0.14	1.05±0.11*#			
Dominant third distal radius	Controls	1.14±0.10	1.20±0.11	11.396	0.002	0.235
	VB players	1.16±0.21	1.36±0.16*#			
Dominant whole radius	Controls	3.92±0.41	4.42±0.47#	12.022	0.001	0.245
	VB players	4.53±0.62*	5.43±0.82*#			
Nondominantultradistal radius	Controls	0.78±0.09	0.86±0.10#	2.260	0.141	0.058
	VB players	0.82±0.15	0.94±0.14#			
Nondominant third distal radius	Controls	1.13±0.12	1.20±0.15#	7.330	0.010	0.165
	VB players	1.11±0.15	1.26±0.17#			
Nondominant whole radius	Controls	3.93±0.47	4.39±0.54#	11.243	0.002	0.233
	VB players	4.28±0.68	5.12±0.69*#			
Lumbar spine L2-L4	Controls	21.70±3.50	24.60±3.90#	8.199	0.007	0.181
	VB players	25.30±3.80	30.60±5.40*#			
Femoral neck	Controls	2.55±0.68	2.69±0.60	8.287	0.007	0.183
	VB players	3.17±0.90	3.89±1*#			
Trochanter	Controls	5.37±1.29	6.27±1.47#	8.587	0.006	0.188
	VB players	6.85±1.77	8.40±2.10*#			
Total hip	Controls	19.70±2.70	21.90±2.90#	7.506	0.009	0.169
	VB players	22.70±4	26.40±5.10*#			

**VB players**, volleyball players;

* Significantly different from Controls at p<0.05;

^#^ Significantly different from Baseline at p<0.05

#### Follow-up measurements

**Fig 1:**
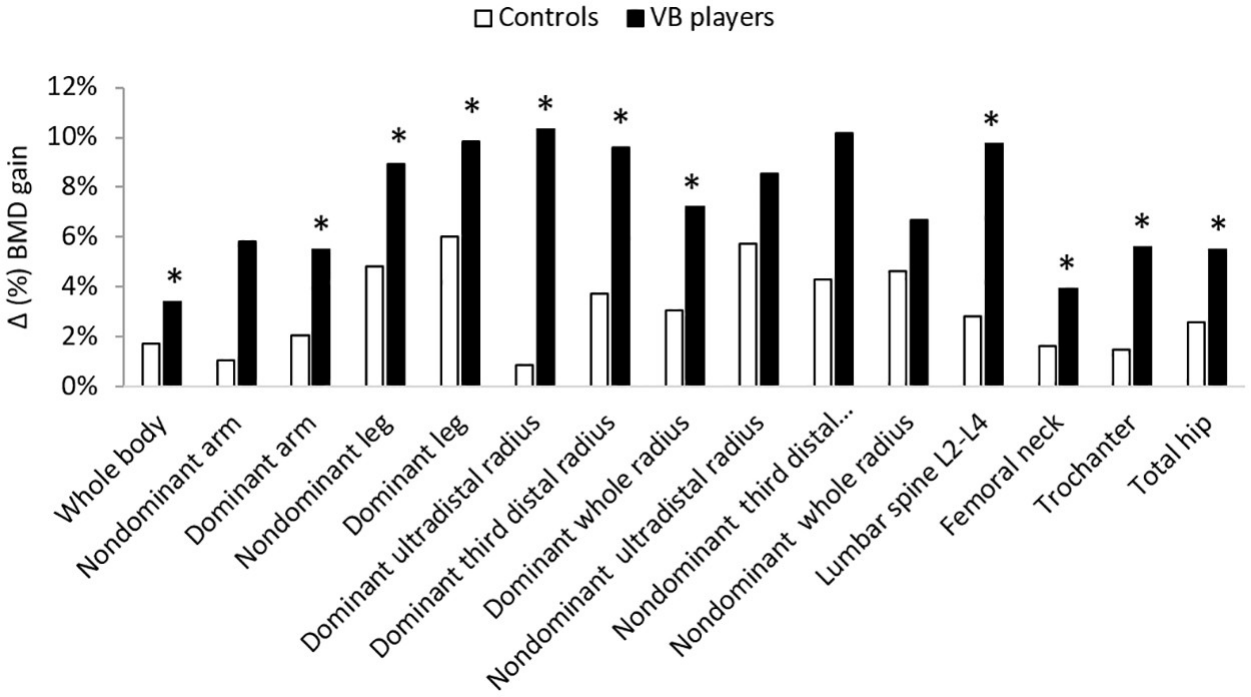
Δ (%) BMD gain at different sites after 1-yr follow-up for controls and volleyball players. * Significantly different from Controls at p<0.05. Δ (%) BMD gain between controls and volleyball players was evaluated after 1-yr follow-up by dual-photon absorptiometry X-rays by DXA at the whole body; lumbar spine (L_2_-L_4_); femoral neck of the dominant leg and dominant and nondominant radius. Volleyball players gained more BMD in all measured sites (p<0.05) except in nondominant arm and radius.

Volleyball players were found to gain more BMD in whole body (4.5% *vs* 1.7%; p = 0.014), both nondominant and dominant arms (5.8% *vs* 1.1% p = 0.005, and 6% *vs* 2.1%; p = 0.003, respectively), both nondmoninat and dominant legs (9% *vs* 4.8%; p = 0.005 and 10.7% vs 6% p = 0.0025; respectively), dominant ultradistal radius (10.4% v*s* 0.9%; p = 0.005), dominant third distal radius (9.6% vs 3.71%; p = 0.023), dominant whole radius (7.4% *vs* 3.1%; p = 0.017), lumbar spine L2-L4 (9.9% *vs* 2.8%; p = 0.004), femoral neck (4.7% *vs* 1.6%; p = 0.034), trochanter (6% *vs* 1.5%; p<0.001) and total hip (6.1% *vs* 2.6%; p = 0.006) (Fig 1).

**Fig 2:**
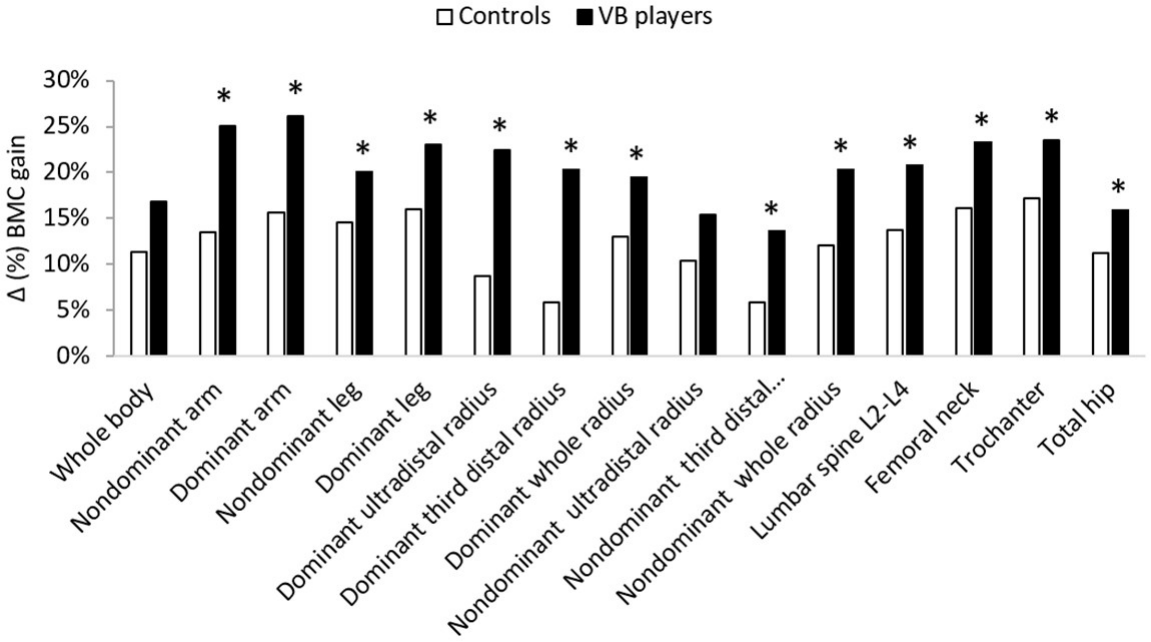
Δ (%) BMC gain at different sites after 1-yr follow-up for controls and volleyball players. * Significantly different from controls at p<0.05. Δ (%) BMD gain between controls and volleyball players was evaluated after 1-yr follow-up by dual-photon absorptiometry X-rays by DXA at the whole body; lumbar spine (L_2_-L_4_); femoral neck of the dominant leg and dominant and nondominant radius. Volleyball players gained more BMC in all measured sites (p<0.05) except in whole body and nondominant ultradistal radius.

For BMC, volleyball players gained more BMC in both nondominant and dominant arms (25.1% *vs* 13.4%; p = 0.003, and 26.1% *vs* 15.6%; p<0.001 respectively), both nondominant and dominant legs (20.2% *vs* 14.5%; p = 0.004 and 23% *vs* 16%; p = 0.004, respectively), dominant ultradistal radius (22.4% *vs* 8.7%; p = 0.002), dominant third distal radius (20.9% v*s* 5.9%; p = 0.001), dominant whole radius (20% *vs* 13%), nondominant third distal radius (14.5% *vs* 5.9%; p = 0.001), nondominant whole radius (21.1% *vs* 12%; p = 0.002), lumbar spine L2-L4 (21.1% *vs* 13.7%; p = 0.007), femoral neck (25.9% vs 8.7%; p = 0.007), trochanter (23.5% *vs*17.1%; p = 0.006), and total hip (16.3% *vs* 11.3%; p = 0.009) than controls (Fig 2).

A non significant difference was found between the 2 groups in nondominant forearm Δ (%) BMD, and in whole body and nondominant ultradistal radius Δ (%) BMC (Figs 1 and 2).

A close correlation was observed between the increment (Δ) of whole body lean mass and increased (Δ) BMD and BMC in whole body (r = 0.43, p<0.01, r = 0.73, p<0.001; respectively), lumbarspine (r = 0.54, r = 0.61, p<0.001; respectively), trochanter (r = 0.46, p<0.01, r = 0.35, p<0.05; respectively), and total hip (r = 0.53, p<0.01, r = 0.6, p<0.0001; respectively) (Table 4).

**Table 4:** Relationship between (∆) increment in whole body LM, and (∆) increased BMD and BMC at different sites.

Whole body LM	r
Whole body BMD	0.43**
Whole body BMC	0.73***
Lumbar spine L2-L4 BMD	0.54***
Lumbar spine L2-L4 BMC	0.61***
Trochanter BMD	0.46**
Trochanter BMC	0.35*
Total hip BMD	0.53**
Total hip BMC	0.6***

* Significant at p<0.05;

** Significant at p<0.01;

*** Significant at p<0.001

## Discussion

The main findings of the present study indicate that pubescent boys (Tanner stage 2), who practiced volleyball for 4–6 hours a week, increased their bone mass, represented by BMD and BMC accrual, more than their non-physically active matched counterparts over 1-yr period. This indicates a positive effect of regular weight-bearing activities on bone mass during growth.

At baseline, our volleyball players were taller, heavier and had higher lean mass at whole body than control subjects. This finding explained the differences of morphologies betweenour volleyball players and control subjects. This result patially agree with previous studies showing higher height, weight and lean mass among prepubescent boys practicing volleyball [17], basketball [14] and judo [30] than their non-physically active matched peers, and contrasts others among handball [31] and soccer players [32]. Contrary to what is known, that the proportion of body fat is usually lower in athlets than in non active subjects [33], our young volleyball players were found to have higher fat mass than controls at baseline. However, others have also reported an increase in fat mass in children associated with increased physical training [34, 35]. Thus, volleyball’s higher fat mass could be explained by an increased food intake accompanying the increased training.

For bone measurements, after adjudting for heigh, weigh and whole body lean mass, the present study did not show anyeffects of volleyball practice at baselinein all measured sites BMD; whearas, BMC was higher in volleyball players only in whole body, dominant arm, both dominant and nondominant leg, and dominant whole radius than controls (p<0.05). Our results contrasts with those of Chaari el al. who found a higher BMC in all measured sites (whole body, lumbar spine, total hip and radius) expect in both right and left third distal radius among prepubertal volleyball players, with a high training level compared to controls [17]. This lack in BMC acquisition in our players could be explained by their lower duration of training (4–6 hours per week vs. 6–8 hours per week).

At follow-up, our volleyball players gained significantly more lean mass in whole body than controls (14.6% vs 11.9%; p<0.05), whereas fat mass in whole body (which was higher in baseline in volleyball players) remains similar between the two groups. Similar findings were reported by Vincenty-Rodgriguez et al. among prepubertal soccer players who gained more lean mass, and maintained their percentage of body fat over 3-yr period compared to controls [36], whereas, zouch el al. (2015) reported no difference in the percentage of change in lean mass between controls and soccer players over 3-yr [32]. Thus, our results support the notion that sports participation may influence body composition by increasing lean mass [37].

No significant differences in dietary calcium intake between the volleyball players and controls were found. Although, the means calcium intake in our study (approximately 750 mg/day), was lower than the official Institute of Medicine (IOM) recommendations (1300 mg/day), could be considered sufficient for optimal skeleton mineralization.

Our study suggested that 4 to 6 h of volleyball practice per week for 1-yr is efficient on bone accretion in boys during childhood and adolesence, and that mechanical stress induced by volleyball practice are site specific. Indeed, volleyball players gained more BMD and BMC over 1-yr in both arms and legs, lumbar spine L2-L4, femoral neck, trochanter and total hip than controls. Our results agree with those of Courteix et al. who reported higher BMD gain in all weight-bearing sites over 1-year in prepubescent gymnast girls than controls [38]. Similarly, Zouch et al, in a 1-yr longitudinal study, showed a greater BMC gain in the lumbar spine and both kicking and supporting legs among prepubertal soccer players compared to controls [12]. Vincente-Rodriguez et al. also showed that, over 3-yr of practice, prepubertal soccer players gained twice as much femoral neck and intertrochanteric BMC than controls and increased their femoral neck BMD by 10% and their mean hip BMD by a third more than controls [36]. Our results can be explained by the response of loaded sites to the significant additional amount of mechanical loads induced by volleyball practice. Infact, during repetitive jumping actions and rapid directionnal changes, lowerlimb’s bones are under tensile, compressive, and torsional stress, which produce high strain stimulus, consequently, bones become more resistant to fractures [39].

The greatest differences between the two groups in lower limb were observed in hip region especially in femoral neck and trochanter. Similarly, Bellver et al. showed that elite female volleyball players displayed the highest values of BMD in femoral neck, trochanter and lumbar spine (L1-L4) compared to aquatic sports athlets, soccer players, field hockey players and controls [40]. This could be explained by the greater forces acting on femoral neck and trochanter during volleyball participation.

Δ (%) BMC enregistred by volleyball players in femoral neck and trochanter were 25.9% and 23.5%, respectively. This is important because it has been reported that even a small increment in femoral neck BMD is associated with a high reduction in the risk of hip fracture in older adults [41]. Annual gains BMC and density enregistreted by our volleyball players were lower than those found by zouch et al. among soccer players in most sites [32]. That difference could be attributed to the lengh of the two studies (3-yr vs 1-yr in our study).

Volleyball players exhibited higher % BMD gains and similar % BMC gains in the whole body. These results are in agreement with previous studies [11, 32, 42] reporting higher BMD and a similar BMC in whole body in adolescent male soccer players than controls. This can be explained by the response of BMD, considered as general sites and the response of BMC as specific sites.

According to this study, volleyball practice is also associated with enhancement of forearm’s bone mineralization. This result is an indicator of responsive radius regions to the significant amount of mechanical loads provided by technical actions such as serving, setting, blocking and spiking the ball, and confirming the site specific effects of volleyball practice. Our volleyball players gained more BMC and density in dominant radius, and BMD in nondominant ultradistal and third distal radius than controls. Our results are in partially accordance with those of previous studies among prepubescent judoists [30], volleyball [17], and basketball players [14]. Contrarily, Zouch et al. did not show any significant difference in BMD in arms among soccer players compared to controls [43]. Differences could be explained by the effect of different types of physical training. Indeed, in judo, basketball and volleyball training, forearm undergoes high strains, whereas in soccer, it is rather the lower limbs that are subject to the impacts.

The present study also showed a positive correlation between the increment of whole body lean mass and increased BMC and density (BMD) in whole body, lumbar spine, trochanter and total hip (Table 4). Thus, the mechanical forces thatact on loaded bones may be generated not only by the high reaction forces produced by impact with the ground in jumping, serving, spiking, and blocking, but also by muscular contractions pulling on their bony attachment. This finding agree with previous studies considering lean mass development as the best predictor of bone mass deposition [14, 44, 45].

The strength of the present study is the 12-month longitudinal observation period, which allowed us to have more reliable data on bone mineral acquisition and actual changes in body composition in growing prepubertal volleyball players, compared to cross-sectional design. However, the current investigation has some limitations. First, the sample size is fairly small. Given the well-knownrole of endocrine mechanisms in bone mass acquisition during the growing years, the lack of hormonal and bone markers data is a further limitation of the present study. This data would have reinforced our results. Future studies are needed to compare bone mass acquisition between boys and girls in growing years.

As practical applications, the present study findings reinforce the need for encourage the practice of weight bearing activities such as volleyball in growin children. This could prevent an early lower bone mineral density in this population, providing a reduction in the risk of developing osteoporosis in the future.

In conclusion, our study suggest that 1-yr of volleyball practice lead to an increase of physical fitness, and lean mass, and report an osteogenic effect on regional BMC, and total and regional BMD in pubescent boys, which may reduce the risk of bone fractures throught life.

## Supporting information

S1 Appendix Anthropometric parameters, calcium intake, physical fitness, and pubertal status at baseline and follow-up. BMI, body mass index; CMJ, Countermovement jump; FSH, follicle-stimulating hormone; HJ, Horizontal jump; LH, luteinizing hormone; PAL, physical activity level; SD, standard deviation; SJ, Squat jump; VB players, volleyball players; VO2max, maximum oxygenuptake (mL/kg/min); * Significantly different from Controls at p<0.05; #, Significantly different from Baseline at p<0.05.
(XLSX)

S2 Appendix Mean ± SD BMD (g/cm2) values for controls and volleyball players at baseline and follow-up. VB players, volleyball players; * Significantly different from Controls at p<0.05; # Significantly different from Baseline at p<0.05.
(XLSX)

S3 Appendix Mean ± SD BMC (g) values for controls and volleyball players at baseline and follow-up. VB players, volleyball players; * Significantly different from Controls at p<0.05; # Significantly different from Baseline at p<0.05.
(XLSX)

S4 Appendix Relationship between (∆) increment in whole body LM, and (∆) increased BMD and BMC at different sites. * Significant at p<0.05; ** Significant at p<0.01; *** Significant at p<0.001.
(XLSX)
