# Correction: Volleyball practice increases bone mass in prepubescent boys during growth: A 1-yr longitudinal study

**DOI:** 10.1371/journal.pone.0295160

**Published:** 2024-02-13

**Authors:** Anis Zribi, Hamada Chaari, Liwa Masmoudi, Wajdi Dardouri, Mohamed Ali Khanfir, Elyes Bouajina, Monia Zaouali, Mohamed Zouch

The affiliation for the eighth author is incorrect. Mohamed Zouch is not affiliated with #1 and #3 but with only #3: Department of Sport Sciences and Physical activity, College of Education, University of H’ail, Saudi Arabia.

In the Funding section, the project number from the funder Scientific Research Deanship at the University of Ha’il—Saudi Arabia is listed incorrectly. The correct project number is: RG_191321.
